# Exploring the Patterns of Use and Acceptability of Mobile Phones Among People Living With HIV to Improve Care and Treatment: Cross-Sectional Study in Three Francophone West African Countries

**DOI:** 10.2196/13741

**Published:** 2019-11-13

**Authors:** Phillipe Lepère, Yélamikan Touré, Alexandra M Bitty-Anderson, Simon P Boni, Gildas Anago, Boris Tchounga, Pendadiago Touré, Albert Minga, Eugène Messou, Guillaume Kanga, Serge Koule, Armel Poda, Alexandra Calmy, Didier K Ekouevi, Patrick A Coffie

**Affiliations:** 1 Institut de Santé Globale Université de Genève, Suisse Genève Switzerland; 2 Programme PACCI Abidjan Cote D'Ivoire; 3 Centre Solidarité Actions Sociales Bouaké Cote D'Ivoire; 4 Centre Médical de Suivi des donneurs de sang Abidjan Cote D'Ivoire; 5 Entre de Prise en charge et de Formation à Yopougon-Attié Abidjan Cote D'Ivoire; 6 Centre Intégré de Recherches Biocliniques d'Abidjan Abidjan Cote D'Ivoire; 7 Unité de soins ambulatoires et de conseils Abidjan Cote D'Ivoire; 8 Hôpital de Jour Service des Maladies Infectieuses et Tropicales CHU Souro Sanou Bobo Dioulasso Burkina Faso; 9 Unité Virus de L'immunodéficience Humaine Service des maladies infectieuses Hôpital Universitaire de Genève Genève Switzerland; 10 Centre Africain de Recherche en Epidémiologie et en Santé Publique Lomé Togo; 11 Département Santé Publique Université de Lomé Lomé Togo; 12 Centre Inserm - 1219 Université de Bordeaux Bordeaux France; 13 Département de Dermatologie et d’Infectiologie Unités de Formation et de Recherche des Sciences Médicales Université Félix Houphouët Boigny, Abidjan Cote D'Ivoire; 14 Service des Maladies Infectieuses et Tropicales Centre Hospitalier Universitaire de Treichville Abidjan Cote D'Ivoire

**Keywords:** acceptability, mHealth, PLHIV, West Africa

## Abstract

**Background:**

The use of mobile technology in health care (mobile health [mHealth]) could be an innovative way to improve health care, especially for increasing retention in HIV care and adherence to treatment. However, there is a scarcity of studies on mHealth among people living with HIV (PLHIV) in West and Central Africa.

**Objective:**

The aim of this study was to assess the acceptability of an mHealth intervention among PLHIV in three countries of West Africa.

**Methods:**

A cross-sectional study among PLHIV was conducted in 2017 in three francophone West African countries: Côte d’Ivoire, Burkina Faso, and Togo. PLHIV followed in the six preselected HIV treatment and care centers, completed a standardized questionnaire on mobile phone possession, acceptability of mobile phone for HIV care and treatment, preference of mobile phone services, and phone sharing. Descriptive statistics and logistic regression were used to describe variables and assess factors associated with mHealth acceptability.

**Results:**

A total of 1131 PLHIV—643 from Côte d’Ivoire, 239 from Togo, and 249 from Burkina Faso—participated in the study. Median age was 44 years, and 76.1% were women (n=861). Almost all participants owned a mobile phone (n=1107, 97.9%), and 12.6% (n=140) shared phones with a third party. Acceptability of mHealth was 98.8%, with the majority indicating their preference for both phone calls and text messages. Factors associated with mHealth acceptability were having a primary school education or no education (adjusted odds ratio=7.15, 95% CI 5.05-10.12; *P*<.001) and waiting over one hour before meeting a medical doctor on appointment day (adjusted odds ratio=1.84, 95% CI 1.30-2.62; *P*=.01).

**Conclusions:**

The use of mHealth in HIV treatment and care is highly acceptable among PLHIV and should be considered a viable tool to allow West and Central African countries to achieve the Joint United Nations Programme on HIV/AIDS 90-90-90 goals.

## Introduction

In 2017, the number of people living with HIV (PLHIV) was estimated to be approximately 36.9 million people worldwide, and the number of new HIV infections was estimated to be 1.8 million [1]*.* In the fight against the HIV epidemic, considerable progress has been made, including an increase in the number of patients on treatment, a decrease in the number of new infections, and a reduction in morbidity and mortality [1]*.* The Joint United Nations Programme on HIV/AIDS (UNAIDS) Fast-Track proposes a framework for achieving a set of ambitious targets for HIV prevention (reduce the number of new infections to <500,000), treatment (reduce the number of AIDS-related deaths to <500,000), and zero discrimination by 2020. As part of this strategy, the world has embraced the 90-90-90 treatment target for the post-2015 era, setting the goal that by 2020, 90% of all PLHIV know their HIV status, 90% of all people with diagnosed HIV infection receive sustained antiretroviral therapy (ART), and 90% of all people receiving ART have durable viral suppression [2].

Today, if treatment is initiated early, adherence is maintained, and quality of care is high, the life expectancy of a person living with HIV is nearly the same as that of a person without HIV [3]. This is true in high-income countries and low-/middle-income countries where some disparities still exist between men and women [4]. As a result, HIV infection is increasingly becoming a chronic disease. Within this paradigm of HIV as a chronic disease, mobile technology can help health systems improve the quality of the HIV treatment cascade. In addition, the World Health Organization (WHO) has endorsed text messaging or short messages service (SMS) interventions for supporting individual-level adherence to ART and for improving linkage of people diagnosed with HIV to HIV-care services [5]. A WHO survey reported that 83% of the Member States used a mobile health (mHealth) initiative for promoting HIV issues [6].

Several pilot projects using mHealth for infectious diseases such as tuberculosis, malaria, and Ebola and for chronic diseases such as hypertension and diabetes have been proven to be feasible and acceptable in the African context, demonstrating the potential of mHealth in contributing to chronic patients’ treatment and care [7-12]. These technologies are also used in HIV treatment and care to improve retention in care and adherence to treatment, which are major challenges to care programs in sub-Saharan Africa, where retention is estimated to be less than 75% at 12 months and 60% at 24 months [13,14]. A systematic review of SMS interventions found that 73% of the studies used “SMS only” interventions, and in only 27% of studies, the use of SMS was associated with other interventions [15]. In sub-Saharan Africa, most interventions using mHealth have mainly used SMS as a way to improve retention of PLHIV in care and adherence to ART. In Cameroon, two interventions among PLHIV used text messages to improve adherence: one used once-per-week motivational messages and the other used four-times-per-week educational messages [16]. Another study in South Africa used an electronic adherence monitoring device and text reminders if patients were late in taking their medications [17]. Text messages reminders for appointments were used to assess retention among PLHIV in Mozambique and were found to improve retention among urban patients and those who newly initiated ART [18].

As part of the standard of care, some interventions were used to improve retention and adherence such as phone calls to patients missing their appointments and home visits in some countries [19]. However, home visits and phone calls, despite their relative effectiveness, constitute a significant financial burden and are not sustainable in the long term [20]. mHealth interventions could decrease this financial burden, especially since mobile phone penetration in sub-Saharan Africa is high and represents an opportunity for the continent [21]. Therefore, there is a need to identify simple, acceptable, and sustainable interventions to improve retention in care and adherence to ART for PLHIV. As a systematic review of mHealth interventions in Africa indicated, successful mHealth interventions would yield positive outcomes if they are accessible, accepted, and low cost while adaptable to the local context [20].

In sub-Saharan Africa, the scientific literature on mHealth has been concentrated in East and Southern Africa, while there is a scarcity of studies in West Africa, especially francophone West Africa countries [22]. A systematic review of mHealth interventions in low-and middle-income countries identified 34 publications from Africa, only two of which were from West Africa including one from a francophone country [22]. Before suggesting mHealth interventions, it is essential to assess the acceptability and feasibility of an mHealth intervention in HIV care and treatment among PLHIV in francophone West Africa. The aim of this study was to explore the social and financial acceptability, which has not been reported yet in Francophone Africa, and the feasibility of an mHealth intervention for the improvement of care among PLHIV in West Africa. We hypothesize that the rates of acceptability would be similar to that found in other regions of Africa.

## Methods

### Study Design and Setting

A cross-sectional study was conducted from September to December 2017 in three francophone West African countries: Côte d’Ivoire, Burkina Faso, and Togo. Six HIV clinics in Côte d’Ivoire, one HIV clinic in Burkina Faso, and one HIV clinic in Togo participated in the study. These centers are reference centers for the three countries, with a significant number of PLHIV in care.

### Participants

Inclusion criteria were age≥18 years, receiving ART for at least 1 year, and receiving follow-up at one of the HIV clinics participating in the study. Participants were selected in each clinic by using a systematic random sampling procedure. Each day of the survey, eligibility criteria for the study were checked by a social worker. Sequential numbers were issued to eligible participants in the order in which they arrived at the HIV clinic. The first eligible participant of the day was included, and a sampling interval of five was applied to select subsequent participants. This procedure was repeated every day in each participating HIV clinic. To avoid possible duplicates, each selected participant received a marked sticker on his medical record.

### Study Size

We hypothesized that mHealth coverage would be around 80% according to the results from previous studies conducted in sub-Saharan Africa [23,24-25]. Considering a relative risk of α=5% and a precision of 5% of the value of the expected acceptability, and taking into account an assumption of 10% of missing data, based on the formula n=z^2^ p(1-p)/α^2^ (with z= confidence level; p=estimated proportion of mHealth coverage in sub-Saharan Africa; α=precision level) for estimation of the sample size, the minimum sample size was estimated at 246 PLHIV on ART in each of the three participating countries for a total of 738 PLHIV [26]. This allowed comparison of the acceptability of mHealth per country.

### Data Collection

A questionnaire was elaborated by a multidisciplinary team of epidemiologists, clinicians, sociologists, social workers, and members of networks of PLHIV. After obtaining informed consent, this standardized questionnaire was administered face-to-face to each participant by trained social workers involved in HIV care and treatment using classical pen and paper material. The questionnaire explored five aspects: sociodemographic characteristics, HIV characteristics, possession of mobile phone, phone sharing, and acceptability (of mobile health). The main outcomes for this study were possession of a phone and acceptability of mHealth (phone calls/text messages).

### Statistical Analysis

The data collected were entered into a Microsoft Access 2013 database (Microsoft Corporation, Redmond, WA) developed for this purpose. Data analysis was performed using STATA (version 11.0; Stata Corp, College Station, Texas). Quantitative variables were described using means and medians, while categorical variables were describing using numbers and proportions. Kruskall-Wallis and Wilcoxon nonparametric tests were used to compare medians for quantitative variables. Chi-square or Fisher exact tests were used to compare categorical variables. Univariate and multivariate regression analyses were carried out to assess factors associated with acceptability of text messages or phone calls to improve health conditions. Regression analyses assessed the factors associated with the acceptability of mHealth on the basis of the towns (Abidjan, Bouaké, Bobo-Dioulasso, and Lomé).

### Ethical Considerations

The protocol for this study was approved by the National Ethics Committee of the Ministry of Health in Côte d’Ivoire, Togo, and Burkina-Faso.

## Results

### Sociodemographic Characteristics

A total of 1131 PLHIV from Côte d’Ivoire (n=643, 56.9%), Togo (n=239, 21.1%), and Burkina Faso (n=249, 22.0%), with a median age of 44 years (interquartile range [IQR]=38-51; Côte d'Ivoire: median=43, IQR=38-50; Burkina-Faso: median=43, IQR: 38-50; Togo: median=44, IQR=37-49; *P*=.04) participated in the study. Among them, 861 (76.1%) were women and about half (n=578, 51.1%) were single. A total of 640 (56.6%) participants had no formal education or a primary educational level and nearly one-third (n=393, 34.7%) could not read or write. Participants from Burkina Faso had the lowest rate of literacy, with 65.1% (n=162) of PLHIV being unable to read and write compared to 25.5% (n=61) from Togo and 26.4% (n=170) from Côte d’Ivoire (*P*<.001). The monthly income of three-quarter of the participants (n=837, 74.0%) was less than 60,000 Franc Communauté Financière Africaine (FCFA) (approximately US $120), which corresponds to the minimum wage in most countries of francophone West Africa. With regard to the HIV treatment status, 785 (69.4%) had been on ART for over 5 years. Other patient characteristics are summarized in Table 1.

**Table 1:** The sociodemographic characteristics of patients in the three participating countries (N=1131).

Characteristic		Côte d'Ivoire (n=643), n (%)	Burkina-Faso (n=249), n (%)	Togo (n=239), n (%)	Total (N=1131), n (%)	*P* value
**Age (years)**						**.02**
	18-34	80 (12.4)	23 (9.2)	38 (15.9)	141 (12.5)	
	35-49	363 (56.5)	160 (64.3)	148 (61.9)	671 (59.3)	
	≥50	200 (31.1)	66 (26.5)	53 (22.2)	319 (28.2)	
**Sex**						**<.001**
	Male	179 (27.8)	40 (16.1)	51 (21.3)	270 (23.9)	
	Female	464 (72.2)	209 (83.9)	188 (78.7)	861 (76.1)	
**Marital status**						**.07**
	Single	335 (52.1)	112 (45.0)	131 (54.8)	578 (51.1)	
	In a relationship	308 (47.9)	137 (55.0)	108 (45.2)	553 (48.9)	
**Level of education**						**<.001**
	No education and primary school	312 (48.5)	194 (77.9)	134 (56.1)	640 (56.6)	
	Secondary school and university	331 (51.5)	55 (22.1)	105 (43.9)	491 (43.4)	
**Literacy**						**<.001**
	Can read or write	473 (73.6)	87 (34.9)	178 (74.5)	738 (65.3)	
	Cannot read or write	170 (26.4)	162 (65.1)	61 (25.5)	393 (34.7)	
**Monthly income (in FCFA^a,b^)**						**<.001**
	<60,000	400 (62.2)	222 (89.2)	215 (90.0)	837 (74.0)	
	60,000-199,999	176 (27.4)	24 (9.6)	20 (8.3)	220 (19.5)	
	>200,000	67 (10.4)	3 (1.2)	4 (1.7)	74 (6.5)	
**Duration of antiretroviral therapy (years)**						**<.001**
	<5	202 (31.4)	59 (23.7)	85 (35.6)	346 (30.6)	
	5-10	207 (32.2)	135 (54.2)	101 (42.3)	443 (39.2)	
	>10	234 (36.4)	55 (22.1)	53 (22.1)	342 (30.2)	

^a^US $1=576.90 FCFA; 60,000 FCFA is the minimum wage in most West African countries.

^b^FCFA: Franc Communauté Financière Africaine.

### Mobile Phone Possession and Access to the Internet

Table 2 summarizes mobile phone possession, mobile internet access, and phone sharing. Overall, the rate of mobile phone possession was 97.9% (n=1107), with a small difference between countries (98.8%, 95.6%, and 97.9% for Côte d’Ivoire, Burkina Faso, and Togo, respectively; *P*=.01). More men owned a mobile phone than women (99.6% vs 97.3%; *P*=.03). With regard to the level of education status, more PLHIV who could read or write possessed phones than PLHIV who could not read or write (99.4% vs 96.7%; *P*=.002). A total of 140 (12.6%) participants shared phones with somebody: 15.4% (n=99) in Côte d’Ivoire, 8.0% (n=20) in Burkina Faso, 8.8% (n=21) in Togo (*P*=.002). Mobile phone sharing was also higher among women and among those with high monthly incomes.

Among PLHIV, 282 (25.5%) had mobile internet access: 30.7% (n=195) from Côte d’Ivoire, 29.9% (n=70) from Togo, and 7.1% (n=17) from Burkina Faso (*P*<.001). Most of those who had a secondary school or university education had better mobile internet access, including young people, women, and those with a higher monthly income (n=208, 42.6%; *P*<.001).

Among mobile phone owners, 33.4% (n=370) owned a smartphone, with a significant difference between countries (73.5%, 3.3%, and 23.2% for Côte d’Ivoire, Burkina Faso, and Togo, respectively; *P*<.001). Among those who owned a smartphone, 83.7% (n=236) had access to mobile internet through mobile phones, with a difference between countries (71.2%, 2.5%, and 26.3% for Côte d’Ivoire, Burkina Faso and Togo respectively; *P*<.001).

**Table 2:** Description of mobile phone possession among patients living with HIV in the three participating countries (N=1131).

Description		Mobile phone possession (n=1107)		Internet access on mobile phone (n=282)		Mobile phone sharing with someone else (n=140)	
		n (%)	*P* value	n (%)	*P* value	n (%)	*P* value
**Country**		N/A^a^	0.013	N/A	<.001	N/A	0.002
	Côte d’Ivoire	635 (98.8)	N/A	195 (30.7)	N/A	99 (15.4)	N/A
	Burkina Faso	238 (95.6)	N/A	17 (7.1)	N/A	20 (8.0)	N/A
	Togo	234 (97.9)	N/A	70 (29.9)	N/A	21 (8.8)	N/A
**Age (years)**		N/A	0.31	N/A	<.001	N/A	0.59
	18-34	136 (96.5)	N/A	61 (44.9)	N/A	20 (14.2)	N/A
	35-49	660 (98.4)	N/A	175 (26.5)	N/A	85 (12.7)	N/A
	≥50	311 (97.5)	N/A	46 (14.8)	N/A	35 (11.0)	N/A
**Sex**		N/A	0.03	N/A	<.001	N/A	0.03
	Male	269 (99.6)	N/A	106 (39.4)	N/A	23 (16.4)	N/A
	Female	838 (97.3)	N/A	176 (21.0)	N/A	117 (83.6)	N/A
**Marital status**		N/A	0.26	N/A	0.38	N/A	0.24
	Single	563 (97.4)	N/A	137 (24.3)	N/A	65 (11.3)	N/A
	In a relationship	544 (98.4)	N/A	145 (26.7)	N/A	75 (13.6)	N/A
**Level of education**		N/A	0.002	N/A	<.001	N/A	0.89
	No education and primary school	619 (96.7)	N/A	74 (12.0)	N/A	80 (12.5)	N/A
	Secondary school and university	488 (99.4)	N/A	208 (42.6)	N/A	60 (12.2)	N/A
**Literacy**		N/A	<.001	N/A	<.001	N/A	0.51
	Can read or write	733 (99.3)	N/A	256 (34.9)	N/A	95 (12.9)	N/A
	Cannot read or write	374 (95.2)	N/A	26 (7.0)	N/A	45 (11.5)	N/A
**Monthly income (in FCFA^b,c^)**		N/A	0.18	N/A	<.001	N/A	. 01
	<60,000	815 (97.4)	N/A	147 (18.0)	N/A	91 (10.9)	N/A
	60,000-200,000	218 (99.1)	N/A	92 (42.2)	N/A	33 (15.0)	N/A
	>200,000	74 (100.0)	N/A	43 (58.1)	N/A	16 (21.6)	N/A
**Duration of antiretroviral therapy (years)**		N/A	0.25	N/A	0.04	N/A	0.34
	<5	335 (96.8)	N/A	97 (29.0)	N/A	48 (13.9)	N/A
	5-10	435 (98.2)	N/A	93 (21.4)	N/A	47 (10.6)	N/A
	>10	337 (98.5)	N/A	92 (27.3)	N/A	45 (13.2)	N/A

^a^N/A: not applicable.

^b^US $1=576.90 FCFA; 60,000 FCFA is the minimum wage in most West African countries.

^c^FCFA: Franc Communauté Financière Africaine.

### Mobile Health Acceptability and Preferred Way of Communication

The overall rate of mHealth acceptability (patients’ acceptability of receiving text messages or phone calls from their physicians) was 98.8% with no variation by country (98.3% in Côte d’Ivoire, 99.1% in Togo, and 100.0% in Burkina Faso; *P*=.08). A total of 386 (34.9%), 708 (63.9%), and 11 (1.0%) patients were willing to receive phone calls only, text messages and phone calls, and text messages only, respectively. Thus, there was a total of 1083 (97.8%) PLHIV who wished to receive phone calls and 708 (63.9%) who wished to receive text messages and phone calls.

There was no statistically significant difference between countries for preference for phone calls (n=618 [57.1%], n=234 [21.6%], and n=231 [21.3%] in Côte d’Ivoire, Burkina Faso, and Togo, respectively; *P*=.47) and for sex (n=260 [24.0%] and n=823 [for men and women, respectively; *P*=.12).

Among participants who preferred text messages, 60.7% (n=430) had a secondary or higher education level, while 39.3% (n=278) had a lower educational level (*P*<.001). Conversely, 56.1% (n=607) of those who preferred phone calls had primary school level or no educational background (*P*=.55).

### Financial Acceptability and Willingness to Pay

Financial acceptability (answers to the question, “How much would you be willing to spend per month, if you could contact your health care worker for your care?”) was also considered, with 50.1% (n=322), 88.7% (n=212), and 92.8% (n=231) of PLHIV in Côte d’Ivoire, Togo, and Burkina Faso, respectively, likely to financially support out-of-pocket expenditures generated by the adoption of a mHealth solution, if any. The options given for the level of monthly contribution each patient was willing to pay for a mHealth intervention varied from 0 to 3000 FCFA (nearly US $0 to $6). A total of 20.1% (n=227) of PLHIV were willing to pay less than 1000 FCFA (nearly US $2), 35.7% (n=404) were willing to pay between 1000 and 3000 FCFA (nearly US $2 to $6), and 11.8% (n=134) were willing to pay more than 3000 FCFA (>US $6). However, there was no significant relationship between financial acceptability and monthly income: 67.4% (n=362) of those who had less than the minimum wage were willing to financially contribute to mHealth costs, and 68.4 (n=201) of those who had more than the minimum wage were also willing to financially contribute to mHealth costs (*P*=.47).

### Factors Associated With Mobile Health Acceptability

In multivariate analysis, factors significantly associated with acceptability of “phone calls” to improve HIV treatment and care were as follows: living in Bouaké, the second town of Côte d’Ivoire (adjusted odds ratio [aOR]=6.24, 95% CI 3.98-9.79; *P*<.001), in Lomé (aOR=0.57, 95% CI 0.37-0.88; *P*<.01), or in Bobo-Dioulasso (aOR=3.06, 95% CI 2.11-4.43; *P*<.001); having a primary school education or no education (aOR=7.15, 95% CI 5.05-10.12; *P*<.001), and waiting over 1 hour before meeting a medical doctor on the appointment day (aOR=1.84, 95% CI 1.30-2.62; *P*=.01; Table 3).

**Table 3:** Factors associated with acceptability of “phone calls” among patients living with HIV in the three participating countries (N=1111).

Factor		Total, n (%)	Bivariate analysis			Multivariate analysis		
			OR^a^	95% CI	*P* value	OR	95% CI	*P* value
**Town**								
	Abidjan (Côte d’Ivoire)	478 (43.0)	1			1		
	Bouaké (Côte d'Ivoire)	150 (13.5)	7.95	5.29-11.95	<.001	6.24	3.98-9.79	<.001
	Lomé (Togo)	234 (21.1)	0.95	0.63-1.40	0.8	0.56	0.36-0.87	0.011
	Bobo-Dioulasso (Burkina Faso)	249 (22.4)	5.28	3.77-7.39	<.001	3.06	2.11-4.43	<.001
**Age (years)**								
	18-34	136 (12.2)	1					
	35-49	659 (59.3)	1.44	0.95-2.17	0.08	N/A^b^	N/A	N/A
	≥50	316 (28.4)	1.53	0.98-2.38	0.06	N/A	N/A	N/A
**Sex**								
	Male	264 (23.8)	1					
	Female	847 (76.2)	1.84	1.34-2.51	<.001	N/A	N/A	N/A
**Marital status**								
	Single	566 (50.9)	1					
	In a relationship	545 (49.0)	1.05	0.82-1.34	0.69	N/A	N/A	N/A
**Level of education**								
	Secondary school and university	480 (43.2)	1			1		
	No education and primary school	631 (56.8)	8.83	6.3-12.22	<.001	7.14	5.04-10.10	<.001
**Time to go to the health center (hours)**								
	≤1	765 (68.8)	1					
	>1	346 (31.1)	1.15	0.88-1.50	0.29	N/A	N/A	N/A
**Time to meet a doctor (hours)**								
	≤1	330 (29.7)	1			1		
	>1	781 (70.3)	1.53	1.16-2.03	0.003	1.84	1.29-2.62	<.001
**Monthly income (in FCFA^c,d^)**								
	<60,000	824 (74.2)	1					
	60,000-199,999	215 (19.3)	0.43	0.30-0.61	<.001	N/A	N/A	N/A
	>200,000	72 (6.5)	0.18	0.08-0.40	<.001	N/A	N/A	N/A

^a^OR: odds ratio.

^b^N/A: not applicable.

^c^US $1=576.90 FCFA; 60,000 FCFA=minimum wage in most West African countries.

^d^FCFA:Franc Communauté Financière Africaine.

## Discussion

### Principal Findings

This study on PLHIV is the first one conducted in francophone sub-Saharan Africa that explores the acceptability of introducing an mHealth solution to better manage HIV treatment and care. In this study, almost all PLHIV reported their acceptability to introduce mHealth into their treatment and care. In addition, acceptability of mHealth was not dependent on sex, age, and level of literacy despite majority of the sample being illiterate. The level of literacy was not a barrier to the acceptability of mHealth, as demonstrated by the fact that almost all indicated their acceptability of mHealth, although the level of literacy conditioned the choice of mHealth services.

Consistent with our findings, previous studies conducted in East and Southern Africa have found the use of mobile phones in the framework of HIV treatment and care acceptable. A 2007 study in South Africa among 300 PLHIV explored the patterns of mobile phone use to improve adherence to ART and reported that 81% had a mobile phone, 99% indicated their acceptability for verbal clinic contact through their mobile phones, and 96% agreed to contact through text messages [27]. The same trend was observed in Kenya, where 99% of the sample supported the use of mobile technology in the management of HIV infection, with calls being the preferred method [28]. Those results suggest that the integration of mobile phone into the treatment and care of PLHIV in sub-Saharan Africa is an opportunity that should be considered.

Our study reported a mobile phone possession rate of 97.9% despite an unequal mobile network coverage across the three countries and confirmed important disparities in access to mobile internet between the countries, as defined by the Global System for Mobile Communications Association mobile connectivity index [21]. In Burkina Faso, a mobile internet-based solution would reach less than 1 of 10 people, while it would reach 3 of 10 people in Côte d’Ivoire and Togo. In these three countries, introducing a non–Web-based solution seems to be the most appropriate intervention. In addition, in a regional context where patients’ fees have been pointed out as a main barrier to access HIV treatment [29,30], our study reported a readiness to pay for better quality health care or at least to support the additional out-of-pocket expenditures generated by the use of their mobile phone to improve the quality of their care, with no significant relationship between financial acceptability and monthly income. Even if our study did not focus on willingness to pay (WTP) as a measure used in the field of health economics, our findings are aligned to the conclusion of a randomized controlled trial carried out in Bangladesh, demonstrating patients’ WTP to benefit from an SMS-based service for managing their type 2 diabetes [31]. In the same country, a previous study already showed patients’ WTP for an improved patient-clinician relationship [32]. However, as there is a scarcity of studies conducted in sub-Saharan Africa in the field of HIV, which target the WTP for digital health services, it seems to be preferable to implement an mHealth solution with a business model that does not increase the user fees, but rather generates benefits to the patients in reducing transportation time, improves waiting time at the clinic, and allows savings on out-of-pocket expenditures.

In our study, participants generally indicated a preference for phone calls, and the acceptability for phone calls was significantly associated with levels of education, such that participants with no education or a primary school level education were more likely to prefer phone calls compared to text messages. This finding of preference for phone calls, and its association with education levels, has been corroborated by other studies in the sub-Saharan region [28,25]. Another study among PLHIV reported that almost all participants indicated their preference for a two-way communication with doctors or nurses, rather than SMS reminders [33]. These findings suggest that mHealth could respond to the need of restoring or strengthening the interface patient-clinician through an improved and personalized relationship. In this way, mHealth could be an appropriate tool to address the issue raised by Tran et al [34] who showed that listening to patients should lead decision makers to improve the quality of health services and decrease the burden of HIV treatment [34]. There is also a need for adaptation of types of communication (text messages or phone calls) to the target PLHIV according to their literacy level. Tailoring text messages interventions to fit with those specific needs of PLHIV is an example of how this adaptation could be implemented. The preference for phone calls could also be explained by several factors including the issue of confidentiality. A qualitative study on PLHIV indicated that although SMS reminders are helpful for treatment adherence, the main concerns are on the delivery mode and the need for confidentiality [35]. This could suggest the importance of using strategies that would protect privacy, ensure confidentiality, and respect human rights for PLHIV.

### Strengths and Limitations

This study was conducted in three countries of West Africa, with a sample of more than 1000 PLHIV from six HIV treatment and care programs, including approximately one-third (34.7%, n=393) who could not read nor write. Findings from this study thus suggest a very good feasibility and acceptability of the use of mHealth in all types of HIV populations. In addition, financial acceptability of mHealth among PLHIV, rarely explored in the literature in West Africa and documented in our study, confer to it an important strength. Furthermore, this study was patient centered, with the involvement of PLHIV from the design and elaboration of the study to the interpretation of the results, as recommended for the design of digital health tool [36]. Most PLHIV organizations of Cote d’Ivoire including the Reseau Ivoirien des organisations de Personnes vivant avec le VIH/Sida (RIP+) and the Coalition des Organisations des Femmes vivant avec le VIH/Sida (COFCI) have been involved in this study since the beginning. In addition, focus groups and interviews were conducted as part of this study among 20 PLHIV and revealed that although messages are highly acceptable, message contents need to be further explored. In fact, PLHIV indicated that contents should be short, highly coded, and customized based on literacy levels.

However, this study has a few limitations. First, it occurred mainly in economic and administrative capitals where mobile network coverage is close to 100% and sharing of mobile phone is limited, even if in these settings, a significant number of people cannot not read or write. If an intervention were to be set up in those cities, further studies would be needed before scaling up to rural and remote areas. Second, only PLHIV already receiving ART were included in the study, of which nearly 70% were stable patients who initiated their treatment over 5 years ago. This could lead to underestimation of the demand for the introduction of an mHealth solution and the willingness to financially contribute, as the targeted population is already adherent and can self-perceive the benefits of the treatment. A bias could have also been introduced with the cross-sectional nature of the study and the fact that recruitment of participants occurred in HIV care and treatment center, systematically including those who are naturally inclined to be retained in care and to adhere to treatment.

Further research, such as qualitative studies, are needed to better understand expectations and perceptions of PLHIV on the contents of text messages or phone calls, especially in terms of confidentiality, privacy, and respect of human rights.

Qualitative studies should also explore health care workers’ perception of the introduction of mHealth into care to assess whether it would be considered a tool to ease their workload or hinder their performance.

Finally, considering that implication of end users is a key factor of success when designing a digital health tool and the need to prevent any potential harm that may result from the use of an mHealth solution in a stigmatizing environment, further qualitative studies shall be conducted in these francophone cities to consider frequency, timing, and content of the SMS in the design of mHealth interventions, as these factors could influence efficacy [36,37].

### Conclusions

Based on a considerable sample size, this study demonstrated that mobile technology is accessible in francophone low-income countries and could be used as a tool to improve the quality of HIV care and treatment. In such a context, mHealth could potentially help francophone West and Central African countries to bridge their treatment gap in the aim to achieve the 90-90-90 goal.

## Abbreviations

ART: antiretroviral treatment
mHealth: mobile health
PLHIV: People Living with HIV
SMS: short messages service
UNAIDS: United Nations Programme on HIV/AIDS
WHO: World Health Organization
